# Pre-Operative Ocular Findings and Long-Term Follow-Up in a Large Cohort of Non-Syndromic Unicoronal Craniosynostosis

**DOI:** 10.3390/jcm12196224

**Published:** 2023-09-27

**Authors:** Parinaz Rostamzad, Yasmin S. Esser, Emily T. C. Tan, Marjolein H. G. Dremmen, Mieke M. Pleumeekers, Sjoukje E. Loudon

**Affiliations:** 1 Department of Plastic and Reconstructive Surgery, Erasmus Medical Center, 3000 CA Rotterdam, The Netherlands; 2 Department of Ophthalmology, Erasmus Medical Center, 3000 CA Rotterdam, The Netherlands; 3 Department of Radiology and Nuclear Medicine, Erasmus Medical Center, 3000 CA Rotterdam, The Netherlands

**Keywords:** unicoronal craniosynostosis, strabismus, refractive errors, amblyopia

## Abstract

(1) Background: Non-syndromic unicoronal craniosynostosis (UCS) is associated with a high prevalence of ocular anomalies. Currently, the etiology of this association remains obscure, however, it is presumed to be primarily attributed to their orbital malformations and/or secondary to craniofacial surgery. We assessed pre-operative ophthalmological examinations of non-syndromic UCS patients and compared them with their postoperative outcomes and long-term follow-up. (2) Methods: A retrospective case series was conducted on medical records of patients with non-syndromic UCS at Sophia Children’s Hospital, Rotterdam. Ophthalmologic examinations were collected at different time periods: T1 (first visit), T2 (<1 year after cranioplasty), and T3 (long-term follow-up at last visit). The McNemar’s test was used for statistical analysis. (3) Results: A total of 101 patients were included, for whom examinations were available at T1 and T3. Patients had a mean age of 2.8 years (±2.7) and 9.5 (±4.9) at T1 and T3, respectively. At T1, 52 patients (51.5%) were diagnosed with strabismus, and 61 patients (60.4%) at T3. Vertical strabismus increased significantly from 23 patients (22.8%) at T1 to 36 patients (35.6%) at T3 (*p* = 0.011). Followed by astigmatism, which increased significantly from 38 (37.6%) at T1 to 59 (58.4%) patients at T3 (*p* = 0.001). T1 was available in 20 patients prior to fronto-orbital advancement (FOA), therefore, a sub-analysis was conducted on these patients, which was followed shortly after FOA at T2. Prior to FOA, strabismus was present in 11 patients (55.0%) and in 12 patients (60.0%) at T2. After FOA, strabismus worsened in two patients. (4) Conclusions: This study showed the high prevalence of ocular anomalies in patients with non-syndromic UCS before and after cranioplasty and at long-term follow-up. The findings of this study show that ophthalmic and orthoptic examinations are an important part of the optimal treatment of patients with non-syndromic UCS.

## 1. Introduction

The neurocranium can be delineated into the cranial base (chondrocranium) and cranial vault (calvaria bones consisting of frontal, parietal, and occipital bones) [1,2]. These calvaria bones are joined together by major sutures constructed from fibrous connective tissue, which allow some flexibility in the skull during growth and development and prevent premature fusion of the bones [1,2]. The growth of the neurocranium and the development of the skull and sutures is a complex process that is influenced by both external and internal factors in an intricate manner that is not yet fully comprehended [3,4]. External factors affecting these sutures involve the growth of the underlying brain and environmental conditions, and intrinsic factors encompass the growth, migration, and differentiation of embryonic cells [3,4]. Premature closure of one or more of these cranial sutures can result in a condition described as craniosynostosis, with a reported prevalence of 5.9 per 10,000 live births worldwide [5]. In 75% of the cases this occurs in isolation, defined as non-syndromic craniosynostosis [6]. However, it can also be part of multiple syndromes including Apert, Crouzon, Saethre–Chotzen, and Muenke syndrome [7]. Unicoronal craniosynostosis (UCS), alternatively referred to as anterior plagiocephaly, ranks as the third most prevalent type of non-syndromic craniosynostosis, following sagittal (scaphocephaly) and metopic (trigonocephaly) synostosis [8]. As a result of the closure of the unicoronal suture, growth of the cranium, maxilla, and facial bones is restricted, leading to retrusion of the forehead at the ipsilateral side and compensatory bossing at the contralateral side, consequently leading to inconsistency in the position of the orbits (orbital dystopia), along with an increased volume of the contralateral orbit [9]. The facial and cranial asymmetry, as well as orbital anomalies, can be corrected through surgery, such as the fronto-orbital advancement (FOA), which is recommended between the ages of 6 and 12 months [10,11] and endoscopic strip craniectomy (ESC) in less severe cases, performed at three months, followed by helmet therapy [12]. The prevalence of ocular anomalies in patients with UCS has been reported to be higher compared to other types of non-syndromic craniosynostosis [13]. The most common ocular anomalies associated with non-syndromic UCS are eye motility disorders (horizontal and vertical strabismus) and refractive errors (astigmatism, hypermetropia, and anisometropia) [13]. Strabismus can occur primarily, but also as a result of craniofacial surgery. The literature reports either an increase (range 21% to 76%), decrease (range 9% to 29%), or no changes at all in the incidence of strabismus after FOA in patients with non-syndromic UCS [14]. Vertical strabismus (hypertropia) is noted to be the most frequently observed form of new-onset strabismus and resolved condition following FOA [14]. Data on the pre-operative prevalence of refractive errors such as astigmatism are limited, while postoperative prevalence has been reported to range from 15% to 92% [14]. In a recent study with a large cohort of patients with isolated UCS, the ophthalmological outcomes of FOA and ESC were compared, revealing an increase in both strabismus and aniso-astigmatism after FOA [15]. However, the pre-operative assessment of most ocular findings was not reported [15]. Currently, data on pre- and postoperative ocular examinations in patients with UCS undergoing cranioplasty is limited, and often small sample sizes are included. Therefore, this study has two primary objectives: firstly, to determine the prevalence of ocular anomalies at first and last examination in all patients with non-syndromic UCS, irrespective of whether patients underwent cranioplasty. Secondly, to assess the pre-operative ophthalmological examinations and compare them with short postoperative outcomes and at long-term follow-up.

## 2. Materials and Methods

### 2.1. Study Design and Participants

A retrospective case series was conducted on patients with non-syndromic UCS between 1994–2023 at Sophia Children’s Hospital in Rotterdam, The Netherlands. Patients were classified as UCS based on clinical examinations and/or cranial CT scans. Furthermore, patients were eligible for inclusion in the study if they had available ophthalmological and orthoptic examinations at the initial and last visit, and if their initial examination had been conducted prior to reaching 18 years of age. All children with confirmed craniosynostosis have been recommended to undergo genetic testing in our center from 2018 onwards [16]. Standard genetic analysis in patients with suspected non-syndromic craniosynostosis includes microarray analysis and the whole exome sequencing craniosynostosis panel. Patients with clinically syndromic craniosynostosis or patients in whom a genetic pathogenic variant was identified were excluded. Patients with missing ophthalmological and/or orthoptic examinations at follow-up were also excluded. The Medical Ethical Committee granted approval for this study, registered under the number: MEC-2022-0309.

### 2.2. Ophthalmological and Orthoptic Examinations

**Figure 1:**
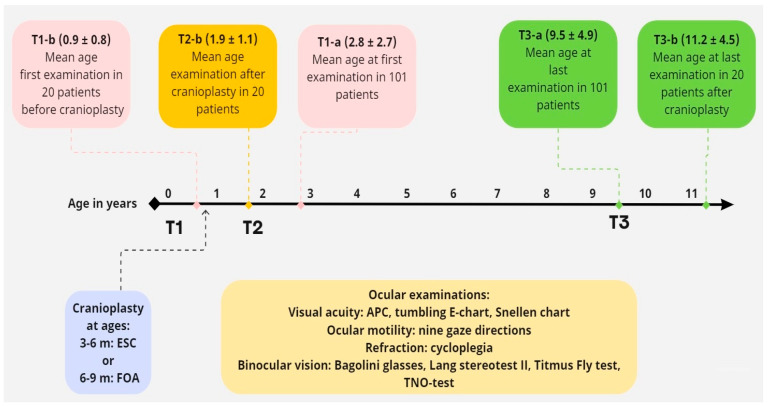
Timeline for the different time periods of ophthalmological measurements.

In this study, the following time periods were identified for the ophthalmological examinations as displayed in Figure 1:

The total study population consisted of 101 patients: T1-a (first ophthalmological examination and T3-a (long-term follow-up examination at last visit).

Subpopulation: 20 patients with available ocular examinations before cranioplasty:

T1-b (first ophthalmological examination before cranioplasty), T2-b (postoperative ophthalmological examination within one year after cranioplasty), and T3-b (long-term follow-up examination at last visit).

The ophthalmological and orthoptic examinations conducted during the initial and final visits were extracted from the electronic medical records. A minimum follow-up duration of one year was ensured between the first and last examination. Examinations consisted of the measurement of strabismus (horizontal, vertical, and alphabetical pattern deviations), using the cover–uncover test. Ocular motility was measured using the nine gaze directions. Esotropia or exotropia was defined as a horizontal deviation of at least 10 prism diopters in primary gaze. Vertical strabismus was defined as a vertical deviation of at least 4 prism diopters in primary gaze. Visual acuity (VA) was measured using the Amsterdam Picture Chart (APC), tumbling E-chart, or Snellen chart depending on the age of the patient. For the analysis of VA, all measurements were converted to the LogMAR scale (Logarithm of the Minimum Angle of Resolution), and for each patient, VA was determined for the better and worse eye. Refractive errors were measured in diopters (D) and were defined as hypermetropia (≥+1.00 D), high hypermetropia (≥+5.00 D), myopia (≥−1.00 D), high myopia (≥−5.00 D), astigmatism (≥±1.00 D), anisometropia (≥1.00 D). Refraction was measured in cycloplegia using 1% cyclopentolate eye drops. Binocular vision was examined with the Bagolini glasses, Lang-stereotest II, Titmus Fly test, or TNO-test, depending on the age of the examination. The degree of binocular vision was defined as not present (negative Bagolini test), poor (positive Bagolini and housefly), moderate (recognition of Titmus Circles 200″–140″, and 100″–40″), good (recognition of TNO plate V 480″–240″, TNO plate VI, or VII 120″–15″). Finally, data on amblyopia and torticollis were determined based on the orthoptic examinations.

### 2.3. MRI Acquisition

MRI brain data were acquired with a 1.5T Unit (General Electronic Healthcare, Chicago, IL, USA), including T1 and T2 sequences. All included MRI scans were analyzed and reported by a radiologist. The MRI images were acquired using the coronal plane.

### 2.4. Statistical Analysis

Descriptive statistics were used for the ocular outcomes and patient characteristics. McNemar’s test was used on paired nominal data for statistical analysis between the first and last examination. A *p*-value of <0.05 was considered statistically significant.

## 3. Results

### 3.1. Study Characteristics

The patient characteristics are presented in Table 1. The initial study population consisted of 148 patients with non-syndromic UCS, of whom 47 patients were excluded due to missing ophthalmological examinations at follow-up. Of the 101 included patients, 64 patients (63.4%) were females. The right suture was closed in 56 patients (55.4%), the left suture in 42 patients (41.6%) and it was unknown in 3 patients (3.0%) (these patients were initially treated elsewhere, and no CT scans were available). In the total population, the mean age was 2.8 years (±2.7 years, range 0–14 years) at the first examination (T1-a), and 10.0 years (±5.0 years, range 2.1–24.8 years) at the last examination (T3-a). Out of the total population, 92 patients (91.1%) underwent FOA, while 5 patients (5.0%) had no history of prior craniofacial surgery due to a mild phenotype and an older age at presentation. For four patients (4.0%), it remained unknown whether cranioplasty was performed as they were treated elsewhere. All patients undergoing FOA were operated on at Sophia Children’s Hospital in Rotterdam. The mean age at FOA was 11.2 months (±6.5 months, range 5–65 months). Ophthalmological examinations before cranioplasty were available in 29 patients (28.7%), of whom 20 had additional post-operative ophthalmological examinations < 1 year after cranioplasty (T2-b).

### 3.2. Prevalence of Ocular Anomalies at First and Last Examination in 101 Patients

The first and last examination in the total population of 101 patients with non-syndromic UCS were compared with ocular anomalies found in a normal Western population, as well as with patients diagnosed with non-syndromic UCS in the literature, presented in Table 2. The mean age at the first examination (T1-a) was 2.8 years (±2.7) and 9.5 (±4.9) at the last examination (T3-a). At first examination, more than half of the patients were diagnosed with strabismus (51.5%). This pattern remained consistent at the last examination, where 61 (60.4%) patients were diagnosed with a type of strabismus. Vertical strabismus was most prevalent in 23 patients (22.8%) at the initial visit, which increased significantly to 36 (35.6%) patients at the last visit (*p* = 0.011). Followed by horizontal strabismus with a vertical component in 19 patients (18.8%) at the initial visit, which increased to 21 patients (20.8%) at the last visit. No significant difference was found for the other types of strabismus between the first and last examination. Between the initial and the last examination, nearly half of the patients, 46 (45.5%), underwent strabismus surgery. Moreover, refractive errors remained notably stable in both the initial and the last examinations, while astigmatism increased significantly from 38 (37.6%) patients to 59 (58.4%) patients at the last examination (*p* = 0.001). At first examination, hypermetropia was present in 82 (81.9%) patients, of whom 3 had high hypermetropia (≥+5.00 D), while 73 (72.3%) patients had hypermetropia at the last examination, of whom 11 had high hypermetropia. In 17 (16.8%) patients where hypermetropia was detected during their initial visit, measurements of refractive errors were not available at follow-up. In total, 52 (51.5%) patients were diagnosed with amblyopia and 44 (43.6%) had torticollis. At the last visit, five patients (5.0%) had a VA of ≥0.5 LogMAR in the worse eye, and four patients (4.0%) had a VA between ≥0.3 ≤0.5 LogMAR in the worse eye.

### 3.3. Sub-Analysis of Ocular Examinations before and after Cranioplasty in 20 Patients

Out of the 29 patients who had available ocular examinations before cranioplasty, 9 patients were excluded from the analysis as they had no follow-up measurements after surgery. A sub-analysis was conducted on the remaining 20 patients who had available ocular examinations before cranioplasty (T1-b), within <1 year after cranioplasty (T2-b), and at the last follow-up (T3-b) as presented in Table 3. All 20 patients were treated by FOA. Mean age at T1-b was 0.9 years (±0.8), 1.9 (±1.1) at T2-b, and 11.2 (±4.5) years at T3-b. Overall, ocular anomalies were found to be highly prevalent before FOA, with a majority of patients being diagnosed with one or more ocular anomalies. Prior to FOA, strabismus was present in 11 (55.0%) patients. Among them, strabismus worsened in two patients after FOA, and both patients developed esotropia. One of these patients had no pre-existent strabismus, while the other patient had pre-existent vertical strabismus which worsened to a combination of esotropia and vertical strabismus. Moreover, four patients developed torticollis (TTC) after FOA, of whom three required additional strabismus surgery to correct the head tilt. Following FOA, all refractive errors remained stable. It is also noteworthy that most ophthalmic parameters remained stable at the last follow-up (T3-b).

### 3.4. MRI Analysis on Eye Muscles in 24 Patients with Non-Syndromic UCS

**Figure 2:**
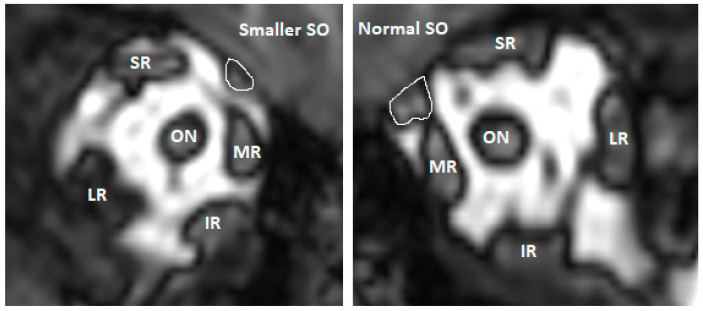
Example of a smaller right superior oblique (SO) muscle in one of the patients with non-syndromic UCS on MRI coronal plane. Abbreviations: SR: superior rectus, MR: medial rectus, LR: lateral rectus, IR: inferior rectus, ON; optic nerve.

**Figure 3:**
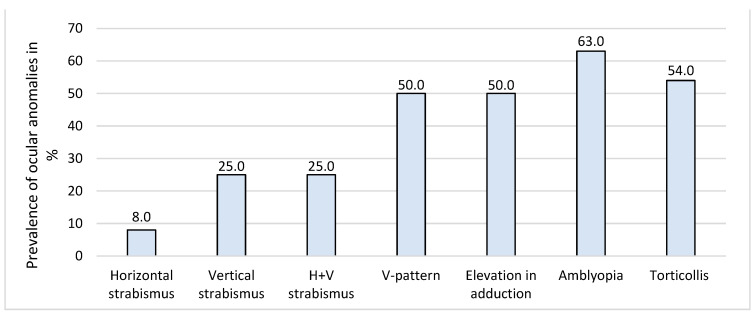
Ocular anomalies in 24 patients (100%) with a reduced volume of superior oblique muscle on MRI at initial visit.

A total of 24 patients had available MRI scans for analysis of ocular muscles, which are presented in Table 4. The images were acquired using the coronal plane. MRI scans before FOA were available for 12 patients (50.0%). All extraocular and oblique muscles were present in all patients. However, the superior oblique muscle was smaller and asymmetric at the ipsilateral side of the closed suture in all 24 patients, with an illustrative example provided in Figure 2 The same pattern of reduced volume size of the superior oblique muscle was seen in patients having MRI scans before and after FOA. Ocular examinations were obtained in all patients with a reduced volume of the superior oblique muscles, presented in Figure 3 at the initial visit. A high prevalence of vertical strabismus (25.0%) and a combination of horizontal + vertical strabismus (25.0%) at the ipsilateral side of the closed suture was seen.

## 4. Discussion

This study is the largest study to report on ocular findings at the initial visit and long-term follow-up in a cohort of 101 patients with non-syndromic UCS. Furthermore, this study is one of the few studies reporting on ocular findings before cranioplasty (FOA), to evaluate the effect of FOA on ocular outcomes in 20 patients. In addition, we confirmed the reduced volume size of the superior oblique muscle on the side of the closed suture on MRI scans in 24 patients. This study showed that the prevalence of ocular anomalies such as strabismus, eye motility disorders, alphabetical pattern deviations, and refractive errors were already high before FOA and remained high after FOA and at long-term follow-up. The findings of this study show that ophthalmic and orthoptic examinations are an important part of the optimal treatment of patients with non-syndromic UCS at a young age.

### 4.1. Strabismus

Strabismus was a common ophthalmic anomaly in this study, which is in line with the literature, where a reported prevalence of 17.0% (95% CI 5.0–33.0, *n* = 186) was found for vertical strabismus in isolated UCS [13]. While the prevalence of strabismus in the normal Western population is found to be much lower, with a reported prevalence of 2.4% (2.1–2.7) for any type of strabismus [17]. Numerous theories have been proposed to explain the etiology of primary strabismus, V-pattern deviation, and eye motility disorders in UCS, which may be due to an excyclorotation of the extra-ocular muscles and muscle cone and displacement of the trochlea on the affected coronal suture, leading to superior oblique underaction and inferior oblique overaction [22,23,24,25,26]. All of these may be the result of the restricted growth of the skull, maxilla, and facial bones, leading to narrow, tall, asymmetrical orbits and a reduced orbital volume on the involved side [9]. Touze et al. quantitatively analyzed MRI scans of the orbits of 15 patients with isolated UCS before cranioplasty and found a significant upward, lateral, and backward displacement of the trochlea, and excyclorotation of the extra-ocular muscles [25]. In our study, an asymmetric and reduced volume size of the superior oblique muscle was observed at the side of the closed suture on the MRI scans of all 24 patients. Half of these patients had either isolated vertical strabismus or a combination of horizontal strabismus with a vertical component, at the ipsilateral side of the closed suture, which might be caused by the weaker action of the superior oblique muscle, due to the reduced volume of the muscle. Moreover, the same study by Touze et al. showed a normalization trend of the excyclorotation of the extra-ocular muscles and the position of the trochlea after FOA in seven patients [25]. However, the effect of this normalization on the clinical outcomes of strabismus and eye motility disorders was not demonstrated due to missing pre- and postoperative ocular examinations [25]. While in our study, pre- and postoperative (FOA), and late follow-up ophthalmological examinations showed that the prevalence of strabismus, eye motility disorders, and alphabetical pattern deviations remained high over time.

Several studies have reported strabismus secondary to craniofacial surgery [10,27,28,29]. More specifically, a higher prevalence of strabismus has been reported after FOA, compared to other types such as distraction osteogenesis [10,28] or ESC [10,15,27]. In a study of 120 patients with isolated UCS, the postoperative ophthalmological outcomes of 60 patients undergoing FOA and 60 patients undergoing ESC were compared [15]. The early postoperative rate of strabismus after FOA was reported to be 31.0%, versus 15.0% after ESC [15], which remained high at late follow-up at 60.0% after FOA versus 35.0% after ESC. [15]. However, as the pre-operative ophthalmological measurements are missing in the study of Elhusseiny et al., it remains unknown if these patients already had a high prevalence of strabismus before surgery [15]. Additionally, an FOA is usually performed in more severe cases, whereas the ESC can be performed in milder cases. Therefore, it may be possible that patients with a more severe form of UCS also have a higher chance of having ocular anomalies, whereas in the milder cases, fewer ocular anomalies may be present. In our study, the prevalence of strabismus before FOA was high at 55.0% (*n* = 20), and only two patients (10.0%) developed strabismus (esotropia) within one year after surgery. Four patients developed torticollis after FOA, which might be due to the young age at examination, or it might be attributed to a surgical complication.

### 4.2. Refractive Errors

Refractive errors, including hypermetropia and astigmatism, had a high prevalence in our study at both the first and last examination. This is in line with the literature, where a prevalence of 35.0% (21.0–51.0, *n* = 102) was found for astigmatism [13]. However, hypermetropia had a lower prevalence of 33.0% (*n* = 27) in the literature, which might be explained due to the threshold (≥1.00 diopter) used in this study, resulting in a higher prevalence of hypermetropia. Compared to the normal population, a prevalence of 12.9% (4.1–21.8) has been reported for astigmatism, and 9.0% (4.3–13.7) for hypermetropia in children [19]. Several hypotheses have been described for the development of astigmatism in patients with isolated UCS [14]. The contralateral fronto-orbital region undergoes compensatory growth, leading to the downward movement of the upper orbital roof [14,30]. This causes compression of the cornea, resulting in a change in its curvature [14,30]. Additionally, astigmatism might develop due to tractional and torsional forces on the cornea due to strabismus [31,32]. In this study, all refractive errors remained stable one year after FOA. Nevertheless, during the last examination, both astigmatism (30.0% to 70.0%) and hypermetropia (85.0% to 95.0%) demonstrated an increase in this study (*n* = 20). This trend aligns with the findings of Elhusseiny et al., who reported an incidence of 72.0% for astigmatism during the last follow-up after FOA [15].

### 4.3. Visual Acuity

Measurement of VA was not available in the majority of the patients at the initial visit (*n* = 65), which may be due to the young age at first examination [33]. At the last examination, VA was 0.00 (±0.8) in the better eye and 0.09 (±0.18) in the worse eye (*n* = 93). This is in line with the literature, where a VA of 0.08 (±0.12, *n* = 28) and 0.10 (±0.15, *n* = 28) was reported for the better and worse eye in children with UCS [18]. Compared to the normal population, a mean VA of 0.0 LogMAR was measured by Polling et al. in a large population-based prospective cohort of 6431 children born between 2002 and 2006 in Rotterdam (Generation R study) [21]. In the same cohort, only six children (0.09%) were classified as having mild visual impairment (0.50 LogMAR) and four (0.06%) had moderate visual impairment (1.00 LogMAR) based on the classification of the World Health Organization [21]. In addition, amblyopia (defined as VA ≥ 0.3 LogMAR in at least one eye, and a difference of 2 LogMAR lines or more) was present in 31 children (0.46%, *n* = 6690) in the study of Polling et al. [21]. Compared to our study, amblyopia was present in 52 patients (51.5%), for which most patients received therapy, resulting in none of the patients having a visual impairment at the last visit in the better eye. At the last visit, only five patients (5.0%) had a VA of ≥0.5 LogMAR in the worse eye, and four patients (4.0%) had a VA between ≥0.3 ≤0.5 LogMAR in the worse eye, of whom most were still under therapy. Therefore, it can be concluded that the majority of the patients with non-syndromic UCS can attain an optimal VA provided that ocular anomalies are screened in a timely manner and appropriately treated when necessary.

### 4.4. Future Research

Patients with UCS show a higher prevalence of ocular anomalies in comparison to other non-syndromic craniosynostosis variants. This disparity is most likely attributed to the orbital malformations causing excyclorotation of the extra-ocular muscles and superior oblique palsy and is less likely due to craniofacial surgery, however, the etiology might also encompass yet unknown genetic causes as contributing factors. In a recent study by Whitman et al., the role of the TWIST-1 gene, linked to Saethre–Chotzen syndrome (syndromal UCS) in mouse models was investigated [34]. Their study revealed that the TWIST-1 gene plays a crucial role in the organization of extra-ocular muscles [34]. Using mouse models, they demonstrated that TWIST-1 deficiency led to disrupted organization of these muscles, resulting in strabismus [34]. Although patients with non-syndromic UCS do not exhibit pathogenic variants in the TCF12 or TWIST-1 genes, nor chromosomal abnormalities, it remains intriguing for future research to explore whether genetic factors contribute to ocular anomalies in non-syndromic UCS patients. Subsequent future studies should incorporate genetic investigations. Additionally, 3D-MRI studies, thoroughly mapping pre- and postoperative ocular muscles and orbits, could offer valuable insight in the quest to uncover causes of ocular abnormalities in patients with non-syndromic UCS, preferably performed in a multi-center prospective manner.

### 4.5. Limitations

Studies with a retrospective design have their limitations. Not all patients could be included due to missing follow-up or incomplete examinations (*n* = 50). Moreover, patients were not always comprehensively examined by the ophthalmologist or orthoptist before cranioplasty, or patients lacked short-term follow-up examinations after cranioplasty, resulting in a reduced number of patients in the sub-analysis of pre-operative ophthalmological findings. In addition, our medical center is a third-line center, and patients often had their follow-ups elsewhere.

## 5. Conclusions

This study showed that the high prevalence of ocular anomalies such as strabismus, eye motility disorders, alphabetical pattern deviations, and refractive errors were already high before FOA, remained high after FOA, and at long-term follow-up. A reduced volume size of the superior oblique muscle on the side of the closed suture was observed in MRI analysis. The findings of this study show that ophthalmic and orthoptic examinations are an important part of the optimal treatment of patients with non-syndromic UCS at a young age.

## Figures and Tables

**Table 1:** Patient characteristics.

Patient Characteristics	Numbers (% or SD)
**Number of patients**	101 (100)
**Sex** (men:female)	37 (36.6):64 (63.4)
**Side suture**	
Right	55 (54.5)
Left	43 (42.6)
Unknown	3 (3.0)
**Type of cranioplasty**	
No surgery	5 (5.0)
FOA ^1^	92 (91.1)
ESC	-
Unknown	4 (4.0)
**Side FOA**	
Both orbital rims	4 (4.0)
Orbital rim one side	86 (85.1)
Unknown which rims	2 (2.0)
Mean age FOA (months)	11.2 (±6.5)
Mean age first examination (y) ^2^	2.8 (±2.7)
Mean age last examination (y)	10.0 (±5.0)

^1^ FOA: fronto-orbital advancement, ^2^ (y): years.

**Table 2:** Prevalence of ocular anomalies in first (T1-a) and last examination (T3-a) in non-syndromic UCS compared with a normal Western population and non-syndromic UCS in the literature.

Ocular Examinations	T1-a (% or SD)	T3-a (% or SD)	*p*-Value T1-T3	Normal Western Population in Literature in % (95% CI)	Non-Syndromic UCS in Literature in % (95% CI, Total *n*)
**Total number of patients**	101 (100)	101 (100)		-	-
**Mean age at examination**	2.8 (±2.7)	9.5 (±4.9)		-	-
**Strabismus**					
Present	52 (51.5)	61 (60.4)	0.078	2.4 (2.1–2.7) ^2^ [17]	-
**Strabismus type**					
Esotropia	4 (4.0)	2 (2.0)	0.625	2.2 (1.1–3.2) ^2^ [17]	10.0 (2.0–21.0, *n* = 120) [13]
Esotropia + vertical	10 (9.9)	8 (7.9)	0.754	-	-
Exotropia	6 (5.9)	2 (2.0)	0.289	1.5 (0.6–2.4) ^2^ [17]	11.0 (5.0–19.0, *n* = 192) [13]
Exotropia + vertical	9 (8.9)	13 (12.9)	0.454	-	-
Vertical	23 (22.8)	36 (35.6)	0.011	-	17.0 (5.0–33.0, *n =* 186) [13]
**Total strabismus surgery**	-	46 (45.5)	-	-	-
Strabismus partly resolved	-	14 (13.9)	-	-	-
Strabismus complete resolved	-	6 (5.9)	-	-	-
New onset strabismus	-	26 (25.7)	-	-	-
**Pattern-deviation**					
A-pattern	7 (6.9)	9 (8.9)	0.727	-	10 (*n* = 20) [18]
V-pattern	37 (36.6)	42 (41.6)	0.532	-	45 (*n* = 20) [18]
**Eye motility disorder**					
Elevation in adduction	55 (54.5)	53 (52.5)	0.855	-	65 (*n* = 20) [18]
Depression in adduction	9 (8.9)	10 (9.9)	1.000	-	-
**Refractive errors**					
Anisometropia	24 (23.8)	27 (26.7)	0.523	-	31.0 (20.0–43.0, *n* = 102) [13]
Astigmatism	38 (37.6)	59 (58.4)	0.001	12.9 (4.1–21.8) ^3^ [19]	35.0 (21.0–51.0, *n* = 102) [13]
Hypermetropia	82 (81.9)	73 (72.3)	0.289	9.0 (4.3–13.7) ^3^ [19]	33.0 (*n* = 27) [20]
Myopia	2 (2.0)	8 (7.9)	0.031	14.3 (10.5–18.2) ^3^ [19]	-
Not examined	13 (12.9)	18 (17.8)	-	-	-
**Visual acuity ^1^**					
Better eye (LogMAR)	0.03 (±0.12)	0.00 (±0.8)	-	0.00 (*n* = 6431) ^4^ [21]	0.08 (±0.12, *n* = 28) [18]
Worse eye (LogMAR)	0.17 (±0.18)	0.09 (±0.18)	-	0.00 (*n* = 6431) ^4^ [21]	0.10 (±0.15, *n* = 28) [18]
Not examined	65	8 (7.9)	-	-	-
**Binocular vision**					
Not present	11 (10.9)	21 (20.8)	0.687	-	-
Poor	2 (2.0)	17 (16.8)	0.375	-	-
Moderate	9 (8.9)	22 (21.9)	0.727	-	-
Good	13 (12.9)	18 (17.8)	0.375	-	-
Not examined	66 (65.3)	45 (44.6)	-	-	-

^1^ All outcomes are presented in numbers, or %, and if available SD or 95% CI, however, for VA outcomes are presented in LogMAR. ^2^ Both children and adults were included, ^3^ only children < 18 were included, ^4^ VA was measured in 6431 normal children from Generation R study, a population-based prospective cohort of children born between 2002 and 2006 in Rotterdam, The Netherlands, only children < 18 were included, if a measurement was not performed or not available a line was introduced.

**Table 3:** Sub-analysis of ocular examinations before FOA (T1-b), after FOA (T2-b), and at last follow-up (T3-b) in 20 patients with non-syndromic UCS.

Ocular Examinations	T1-b (% or SD)	T2-b (% or SD)	T3-b (% or SD)
**Total number of patients**	20 (100)	20 (100)	20 (100)
**Mean age at examination (y)**	0.9 (±0.8)	1.9 (±1.1)	11.2 (±4.5)
**Strabismus**			
Present	11 (55.0)	12 (60.0)	10 (50.0)
**Strabismus type**			
Esotropia	1 (5.0)	2 (10.0)	-
Esotropia + vertical	5 (25.0)	6 (30.0)	2 (10.0)
Exotropia	-	-	-
Exotropia + vertical	3 (15.0)	3 (15.0)	3 (15.0)
Vertical	2 (10.0)	1 (5.0)	5 (25.0)
**Torticollis (TTC)**	1 (5.0)	5 (25.0)	2 (10.0)
**Total strabismus surgery**	-		12 (60.0)
Strabismus surgery for TTC	-	3 (15.0)	-
Strabismus partly resolved	-	-	5 (25.0)
Strabismus complete resolved	-	1 (5.0)	2 (10.0)
New onset strabismus	-	2 (10.0)	3 (15.0)
**Pattern-deviation**			
A-pattern	1 (5.0)	1 (5.0)	1 (5.0)
V-pattern	5 (25.0)	9 (45.0)	10 (50.0)
**Eye motility disorder**			
Elevation in adduction	10 (50.0)	12 (60.0)	12 (60.0)
Depression in adduction	2 (10.0)	3 (15.0)	2 (10.0)
**Refractive errors**			
Anisometropia	5 (25.0)	5 (25.0)	6 (30.0)
Astigmatism	6 (30.0)	6 (30.0)	14 (70)
Hypermetropia	17 (85.0)	17 (85.0)	19 (95.0)
Myopia	-	-	1 (5.0)
Not examined	3 (15.0)	3 (15.0)	-
**Visual acuity ^1^**			
Better eye (LogMAR)	0.03 (±0.12)	0.07 (±0.11)	0.00 (±0.08)
Worse eye (LogMAR)	0.17 (±0.18)	0.07 (±0.11)	0.05 (±0.17)
Not examined	18 (90.0)	17 (85.0)	-
**Binocular vision**			
Not present	2 (10.0)	2 (10.0)	4 (20.0)
Poor	-	-	4 (20.0)
Moderate	-	-	4 (20.0)
Good	-	-	4 (20.0)
Not examined	18 (90.0)	18 (90.0)	4 (20.0)

^1^ All outcomes are presented in numbers, or %, and if available SD or 95% CI, however, for VA outcomes are presented in LogMAR.

**Table 4:** MRI analysis on eye muscles in 24 patients with non-syndromic UCS.

**Number of patients**	24 (100)
**Mean age at MRI (y)**	3.6 (±4.3)
**Side suture**	
Right	16
Left	8
**Extraocular muscles**	
Present	24
**Oblique muscles**	
Present	24
**Reduced volume**	
**m. superior oblique**	
Right eye	16
Left eye	8

## Data Availability

Data are described in Castor electronic database. Links to Castor electronic database could not be made public due to privacy restrictions, approved by the Institutional Review Board of Erasmus Medical Center, The Netherlands.
